# Mutations of p53 decrease sensitivity to the anthracycline treatments in bladder cancer cells

**DOI:** 10.18632/oncotarget.25530

**Published:** 2018-06-19

**Authors:** Sony Pandey, Jennifer Bourn, Maria Cekanova

**Affiliations:** ^1^ Department of Small Animal Clinical Sciences, College of Veterinary Medicine, The University of Tennessee, Knoxville, Tennessee 37996, USA; ^2^ UT-ORNL Graduate School of Genome Science and Technology, The University of Tennessee, Knoxville, Tennessee 37996, USA

**Keywords:** p53 siRNA, bladder cancer, PRIMA-1, AD 198, AD 312

## Abstract

Due to doxorubicin (Dox) cardiotoxicity, the next generation of novel non-cardiotoxic anthracyclines, including AD 312 and AD 198, were synthesized and validated. In this study, we assessed the efficacy and mechanisms of anthracyclines-induced apoptosis and inhibition of cell viability in human bladder cancer cells expressing wild-type (wt) p53 (RT4 and SW780) and mutated (mt) p53 (UM-UC-3, 5637, T-24, J82, and TCCSUP) protein. Anthracyclines inhibited cell viability in tested TCC cells, but were less effective in mt-p53 TCC cells, especially in the drug-resistant J82 and TCCSUP cells. Anthracyclines upregulated the expression of wt p53 protein in RT4 and SW780 cells, but had no effect on expression of mt p53 protein in UM-UC-3, 5637, T-24, J82, and TCCSUP cells. The anthracyclines activated caspase 3/7 and cleavage of PARP in wt-p53 RT4 and SW780 cells, and mt-p53 5637, UM-UC-3, and T-24, but not in mt-p53 J82 and TCCSUP cells. The anthracyclines-induced cleavage of PARP was blocked by p53 siRNA in wt-p53 RT4 cells. Co-treatment of AD 198 with PRIMA-1 significantly inhibited cell viability of mt-p53 J82 cells, but had no effect in wt-p53 RT4 cells. AD 198 blocked c-myc expression in mt-p53 UM-UC-3, 5637, T-24, and J82 cells, however no expression of c-myc was detected in wt-p53 RT4 and SW780 cells. In conclusion, our results demonstrated that the anthracycline-induced resistance in bladder cancer cells positively correlated with *TP53* mutations in the tetramerization domain in J82 and TCCSUP cells. Further, AD 312 and AD 198 are promising chemotherapeutic drugs for bladder cancer, especially in combination with PRIMA-1.

## INTRODUCTION

Bladder cancer is the fourth most common cancer expected to occur in men in the United States in 2018 and is one of the leading causes of cancer deaths predominantly in men [1]. Its high recurrence rates and rapid progression from non-invasive to invasive stages necessitates more effective treatment options [2]. Doxorubicin hydrochloride (Dox) is a chemotherapeutic drug used for the treatment of a wide range of cancers, including bladder cancer [3]. Dox is an anthracycline antibiotic that halts proliferation of dividing cells by intercalating into DNA and inhibiting topoisomerase II-mediated DNA repair mechanisms [4]. Dox also induces cell death by the production of reactive oxygen species, which causes lipid peroxidation and membrane damage in cells [5]. While Dox is a highly effective chemotherapy drug, cardiotoxicity and development of resistance associated with its prolonged use has been observed in cancer patients (Reviewed in [6, 7]). Since the anti-proliferative properties of Dox and cardiotoxicity associated with its use occurs through separate mechanisms, development of effective but non-cardiotoxic anthracyclines has been intensively investigated [8]. The next generation anthracyclines, AD 312 *(N*-Nitrosureidodaunorubicin; Daunomustine^®^, Paradox Pharmaceuticals, Inc.) and AD 198 (N- benzyladriamycin-14-valerate; Benzarubicin^®^, Paradox Pharmaceuticals, Inc.) were synthesized and their efficacy and mechanisms of action were studied [9–11].

AD 312 (Daunomustine^®^) is a hybrid anthracycline with a unique structure that contains the anthracycline ring and a nitrosoureido-alkyl group [11]. The anthracycline ring in AD 312 strongly intercalates with DNA and causes protein-associated DNA strand breaks leading to the inhibition of topoisomerase II activity, a mechanism similar to Dox. The nitrosoureido-alkyl group of AD 312 causes direct DNA strand breaks and crosslinks leading to inhibition of DNA synthesis and repair [11]. Due to this unique bifunctional structure, AD 312 has improved anti-proliferative efficacy as compared to Dox treatment. AD 312 inhibits growth of Dox-sensitive and Dox-resistant murine P388 leukemia cells *in vitro* [12]. Since the Dox-resistant P388 leukemia cells have low topoisomerase II levels [13], their sensitivity to AD 312 is due to activity of the nitrosouredio-alkyl group [14]. In addition to its *in vitro* efficacy, AD 312 inhibits Dox-sensitive and Dox-resistant murine leukemia P388, human ovarian A2780/DOX5, and bladder UCRU-BL13 xenograft tumors in mice without the toxicity observed in Dox-treated mice *in vivo* [12, 15]. In conclusion, AD 312 has dual anti-tumor properties, lower toxicity, and increased efficacy compared to Dox *in vivo*.

AD 198 (Benzarubicin^®^) is also a hybrid anthracycline with a unique structure that contains an N-benzyl ring and a valerate group. Similar to AD 312, the unique structure of AD 198 provides bifunctional anti-tumor properties, as well as novel mechanisms to evade drug-induced resistance [8]. The 3’-N-benzyl ring and the 14-O–valerate group in the structure of AD 198 increase its lipophilic and cell penetrating capacity, its localization in the cytoplasm, and its binding to the regulatory domain of protein kinase C (PKC) resulting in induction of cell apoptosis [8, 16, 17]. Hydrolysis of the 14-O-valerate side chain of AD 198 yields its conversion to N-benzyladriamycin (AD 288), which inhibits topoisomerase II catalytic functions and inhibits DNA synthesis and repair [8]. AD 198 induces apoptosis in canine bladder TCC cancer cells thorough the PKC-δ dependent pathway and the activation of p38 MAPK through the phosphorylation of transcription factors, including cAMP response element binding (CREB) protein and activating transcription factor 2 (ATF2) [18]. AD 198 induces apoptosis in PKC-δ-independent mechanisms through the suppression of oncoprotein c-myc in multiple myeloma and lymphoma cells [19]. In addition, AD 198 is non-cardiotoxic, and instead has cardio-protective effects in rats and mice *in vivo* [11, 20, 21]. Combined with its superior anti-tumor activity, lower systemic toxicity, and cardio-protective effects [11], AD 198 might be a better treatment option for patients with acquired Dox-resistant cancers, especially for patients with underlying heart conditions.

The wild-type p53 protein, which is encoded by the *TP53* gene, plays an important role as a tumor suppressor in regulation of cell cycle arrest, DNA repair, and apoptosis. A recent comprehensive study investigating 131 invasive urothelial bladder carcinomas identified inactivated p53 through *TP53* gene mutations in 49% of tested samples, thus, highlighting its relevance in diagnosis and treatment management of bladder cancers [22]. The association between p53 overexpression, mutations, and drug resistance has been reported in bladder [23], breast [24, 25], ovarian [26], and other types of cancer [25, 27–29]. The majority of *TP53* mutations appears within a DNA-binding domain (DBD) [25, 30, 31], however mutations in the tetramerization domain (TMD) abolishes its DNA-binding activity [32]. Mutations of *TP53* are more common in high-grade invasive bladder cancers [33, 34]. Since chemotherapeutic drugs act through p53-dependent apoptotic mechanisms, high-grade tumors that have *TP53* mutations are often resistant to chemotherapy treatments. Thus, re-activation of mutant p53 in those tumor cells may restore p53 tumor-suppressor function and sensitize mt-p53 cells to chemotherapy treatments [28]. PRIMA-1 (P53 Reactivation and Induction of Massive Apoptosis-1) is a small molecule drug that restores the transcriptional functions of p53 in cells with mutated p53 [35, 36]. PRIMA-1 alone or in combination with other drugs are currently investigated for treatment of p53 mutant prostate, ovarian, and other types of cancer [37].

In this study, we compared the efficacy and mechanisms of Dox, AD 312, and AD 198 treatments in inhibition of human bladder TCC cells expressing wild-type and mutated p53 protein. In addition, we evaluated the efficacy of these anthracyclines in combination with PRIMA-1 treatment to induce apoptosis in the chemo-resistant bladder cancer cells *in vitro*.

## RESULTS

### Mutation of p53 protein decreased sensitivity to anthracycline treatments in human bladder TCC cells

**Figure 1:**
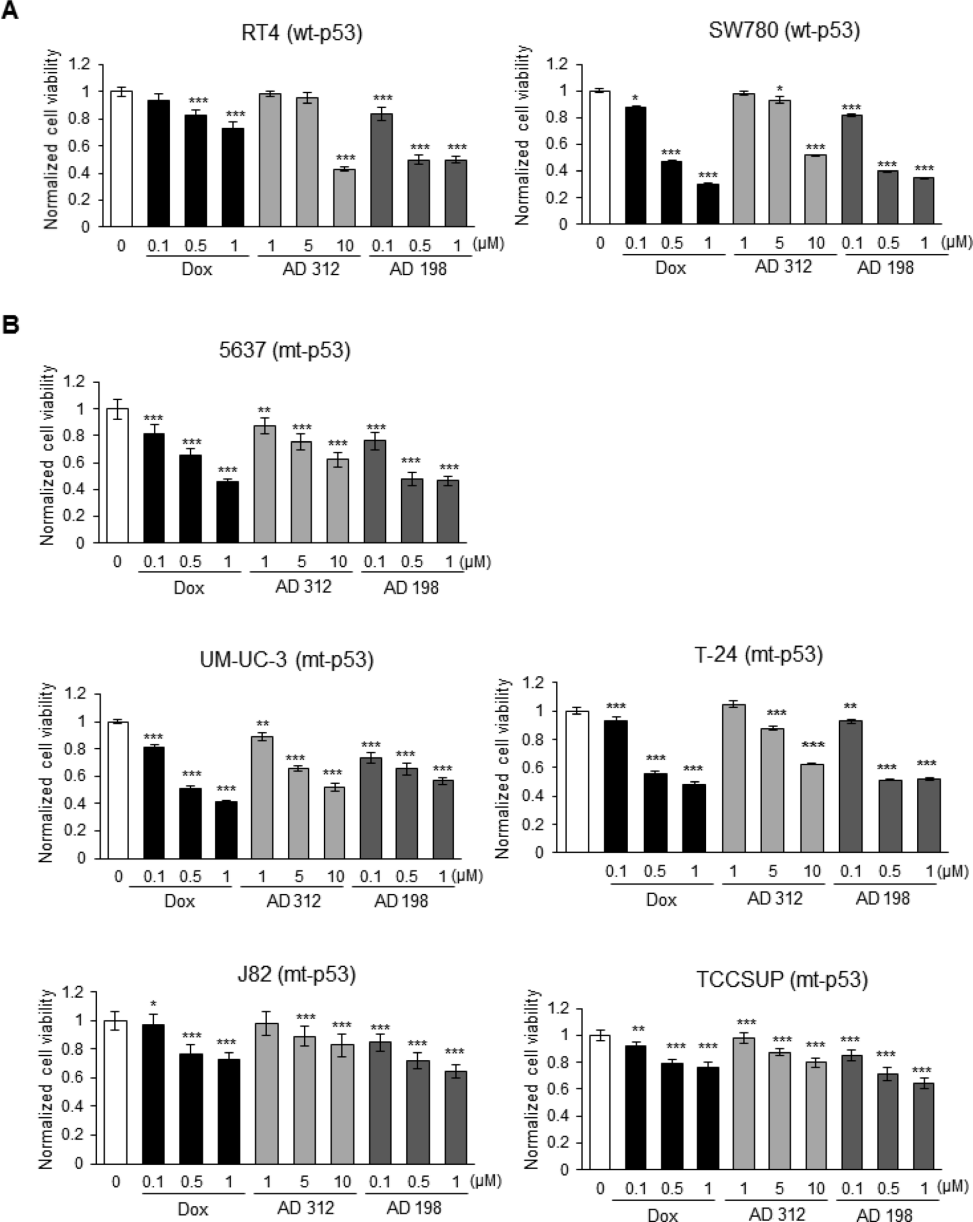
Dox, AD 312, and AD 198 treatments inhibited viability of bladder TCC cells (**A**) Human wt-53 RT4 and SW780 cells, and (**B**) human mt-p53 5637, UM-UC-3, T-24, J82, and TCCSUP cells were treated with 0.1 µM, 0.5 µM, and 1 µM of Dox or AD 198, or 1 µM, 5 µM, and 10 µM of AD 312 for 48 hours and cell viability was assessed by MTS assay. Tested drugs decreased cell viability of bladder TCC cells in a dose-dependent manner; however, high grade mt-p53 J82 and TCCSUP cells were the most resistant to the anthracycline treatments. AD 198 was the most effective anthracycline in the inhibition of cell viability of tested bladder TCC cells. Values shown are the means ± S.E. of four replicates of two independent experiments of normalized cell viability of drug-treated groups to the DMSO-treated (control) groups. Statistical analyses were performed using the Student’s two tailed paired *t*-test and significance was determined at ^*^p ≤ 0.05, ^**^p ≤ 0.01, and ^***^p ≤ 0.001.

Human wt-p53 RT4 (Grade I) and SW780 (Grade I), and mt-p53 5637 (Grade I), UM-UC-3 (Grade III), T-24 (Grade III), J82 (Grade III), and TCCSUP (Grade IV) bladder TCC cell lines (Table 1) were treated with 0.1, 0.5, and 1 µM Dox and AD 198, and 1, 5, and 10 µM AD 312 for 48 hours. Dox at 0.5 µM decreased cell viability by 53% in SW780 cells, but in RT4 cells, only a 17% and 27% inhibition in cell viability was detected by 0.5 µM and 1 µM Dox treatments, respectively. A high dose of AD 312 (10 µM) significantly decreased cell viability by 57% and 48% in wt-p53 cell lines, RT4 and SW780 (Figure 1A), respectively, while AD 198 at a low dose of only 0.5 µM decreased cell viability by more than 50% as compared to control. The mt-p53 bladder TCC cell lines 5637, UM-UC-3, and T-24 (Figure 1B and Table 2) showed a dose-dependent inhibition of cell viability by tested anthracyclines. However, high grade mt-p53 J82 and TCCSUP cells (Figure 1B and Table 2) with mutations in the TMD of *TP53* were resistant to all anthracycline treatments as compared to wt-p53 or other tested mt-p53 cells.

**Table 1:** *TP53* gene mutation status in tested bladder TCC cells

TCC cell line	Grade	*TP53* mutation status	Domain	Gene Sequence	Protein Sequence	Function of mutated *TP53* in cancer
**RT4**	I [63, 43]	wt	-	-	-	-
**SW780**	I [63, 43]	wt	-	-	-	-
**5637**	I [63] or II [43]	mt/Exon 8	DBD DBD	c.733 G>A c.839 G>C	p.G245S [46] p.R280T [42, 43, 46]	EMT, anchorage- independent growth [30, 47] Cell survival, anchorage-independent growth/Anoikis Proliferation [30, 45]
**UM-UC-3**	III [43, 63]	mt/Exon 4	DBD	c.338 T>G	p.F113C [42, 43, 46]	N/A
**T-24**	III [43, 63]	mt/Exon 5	DBD	c.378 C>G	p.Y126 [43, 46]	N/A
**J82**	III [43, 63]	mt/Exon 8 mt/Exon 8 mt/Exon 9	DBD DBD TMD	c.811 G>A c.820 G>T c.960 G>C	p.E271K [42, 43] p.V274F [42, 43] p.K320N [42, 43]	N/A Cell survival, Drug resistance/Avoidance of Cell Death [30, 49, 50] Non-specific DNA binding [48]
**TCCSUP**	IV [43, 63]	mt/Exon 10	TMD	c.1045 G>T	p.E349 [43, 46]	Cytoplasmic retention and loss of transactivation function [51]

DBD: DNA binding domain; TMD: Tetramerization domain.

**Table 2:** IC_50_ (µM) values for tested bladder TCC cells treated with Dox, AD 312, and AD 198

TCC cell line	IC_50_ (µM) Dox	IC_50_ (µM) AD 312	IC_50_ (µM) AD 198
**RT4**	1.84	10.21	0.82
**SW780**	0.63	11.62	0.61
**5637**	0.86	13.10	0.75
**UM-UC-3**	0.72	9.74	1.08
**T-24**	0.81	13.2	0.82
**J82**	1.65	28.37	1.35
**TCCSUP**	2.0	24.1	1.34

IC_50_ values for Dox, AD 312, and AD 198 treatments for tested cell lines were calculated as shown in Table 2. Lower IC_50_ values for AD 198 compared to Dox and AD 312 treatments indicates that AD 198 was the most effective in inhibiting cell viability of tested TCC cells, except UM-UC-3 cells (Table 2). Similar IC_50_ values of AD 198 and Dox treatments were detected in tested TCC cells, while 10-fold higher doses of AD 312 were required as compared to Dox or AD 198 treatments to achieve similar inhibition of cell viability (Table 2).

### Dox, AD 312, and AD 198 treatments increased the expression of p53 protein in wt-p53 human bladder cancer cells

**Figure 2:**
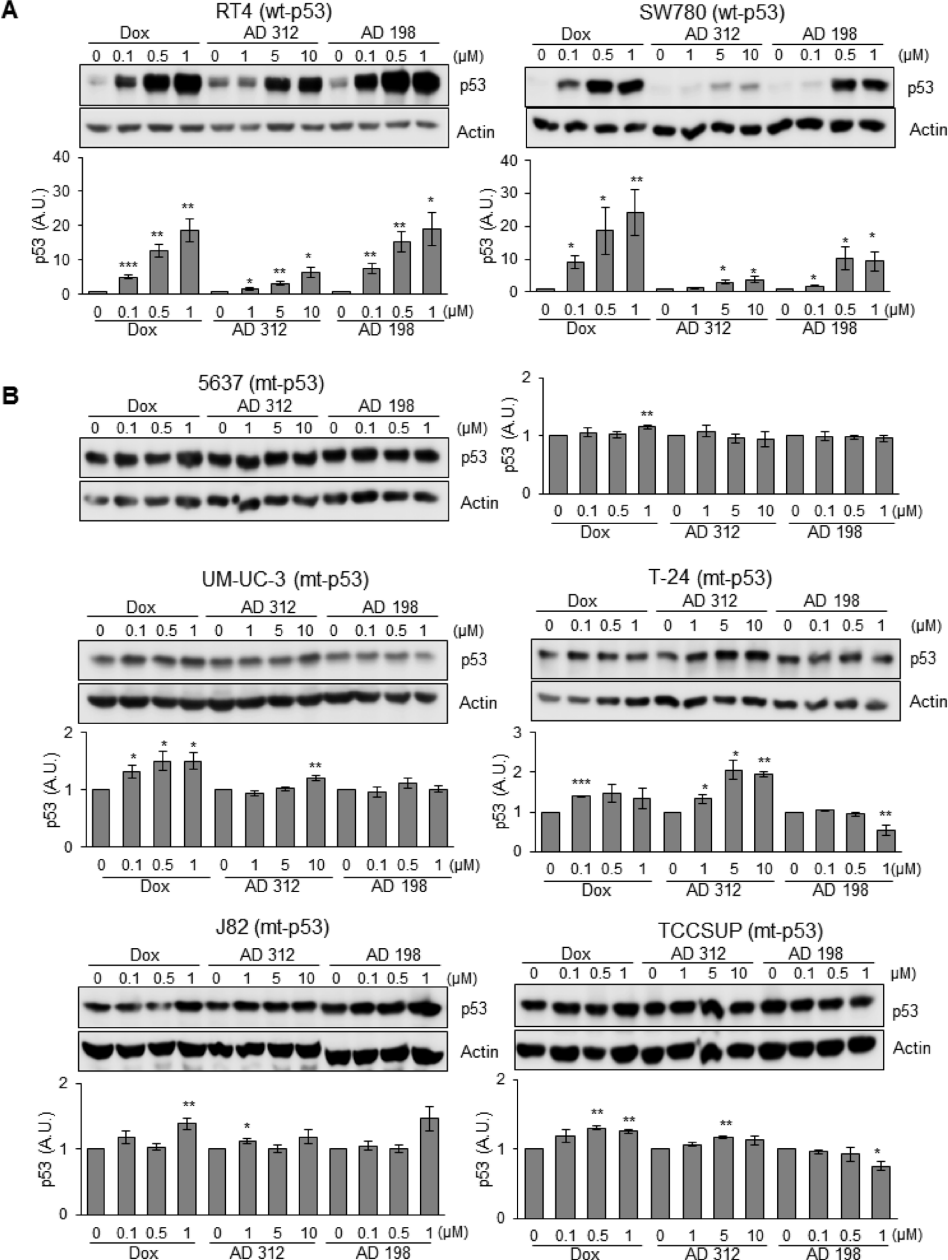
Dox, AD 312, and AD 198 treatments increased p53 protein expression in wt-p53 bladder TCC cells Human bladder TCC cells were treated with 0.1 µM, 0.5 µM, and 1 µM of Dox or AD 198 or 1 µM, 5 µM, and 10 µM of AD 312 in serum free media for 24 hours and levels of p53 protein expression were detected by WB analysis. Actin was used as a loading control. (**A**) Significant increase in p53 protein levels were detected in wt-p53 RT4 and SW780 cells after anthracycline treatments in a dose-dependent manner. (**B**) Slight to no change in the expression of p53 protein levels were detected in the mt-p53 5637, UM-UC-3, T-24, J82, and TCCSUP cells after either anthracycline treatment. Densitometry analysis of normalized p53 protein levels to actin and relative to the DMSO-treated (control) groups are shown as means ± S.E. of three readings of two independent experiments. Statistical analyses were performed using the Student’s two tailed paired *t*-test and significance was determined at ^*^p ≤ 0.05, ^**^p ≤ 0.01, and ^***^p ≤ 0.001.

To elucidate the effects of Dox and AD treatments on the expression of p53 protein, cells were treated with 0.1, 0.5, and 1 µM of Dox and AD 198 and 1, 5, and 10 µM of AD 312 for 24 hours. High dose of Dox, AD 312, and AD 198 treatments elevated p53 expression by more than 18-, 6-, and 19- fold, respectively in wt-p53 RT4 and by more than 24-, 4-, and 9- fold, respectively in SW780 cells as shown in Figure 2A. However, neither Dox, AD 312, nor AD 198 treatments significantly increased (less than 2-fold) the levels of p53 expression in all tested mt-p53 5637, UM-UC-3, T-24, J82, and TCCSUP cells (Figure 2B). The basal levels of p53 protein expression in tested seven human bladder cancer cells were shown in Supplementary Figure 1. TCCSUP cells has a truncated p53 protein, which was detected at lower molecular weight.

### Dox-, AD 312-, and AD 198-induced apoptosis through activation of caspase-3/7 and cleavage of PARP

**Figure 3:**
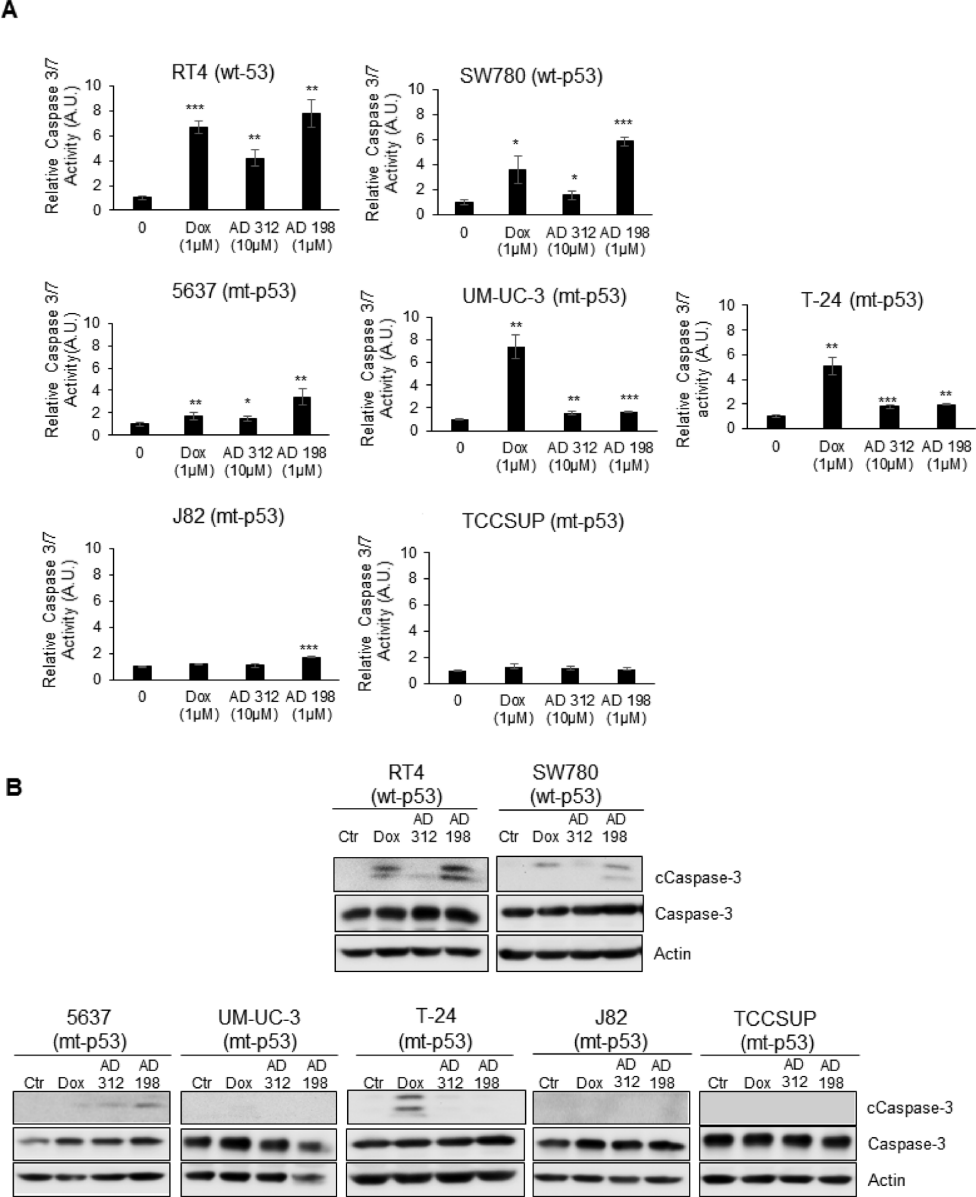
Dox, AD 312, and AD 198 treatments increased caspase-3/7 activity and cleaved caspase-3 protein expression in wt-p53 bladder TCC cells The wt-p53 RT4 and SW780 cells, and the mt-p53 5637, UM-UC-3, T-24, J82, and TCCSUP cells were treated with 1 µM Dox, 10 µM AD 312, and 1 µM AD 198 for 24 hours. (**A**) The activities of caspase 3/7 were measured by Caspase-Glo3/7 luminescence assay. A significant increase in caspase 3/7 activities by Dox, AD 312 and AD 198 treatments were detected in wt-p53 RT4 and SW780 cells. Only moderate upregulation of caspase 3/7 activities by Dox, AD 312 and AD 198 were observed in mt-p53 5637 cells. Dox treatment significantly increased caspase 3/7 activity in mt-p53 UM-UC-3 and T-24 cells. Low caspase 3/7 activities were detected in mt-p53 J82 and TCCSUP cells. Data shown here are mean ± S.E. of three replicates of two independent experiments of normalized caspase activities of drug-treated groups to the DMSO-treated (control) groups. Statistical analyses were performed using the Student’s two tailed paired *t*-test and significance was determined at ^*^p ≤ 0.05, ^**^p ≤ 0.01, and ^***^p ≤ 0.001. (**B**) Levels of expressed cleaved and total caspase-3 proteins were detected by WB analysis. Actin was used as a loading control. Dox, AD 312, and AD 198 treatments increased cleavage of the caspase-3 in wt-p53 RT4 and SW780 cells. AD 198 increased cleavage of caspase-3 in mt-p53 5637 cells, and Dox increased cleavage of caspase-3 in mt-p53 UM-UC-3 and T-24 cells. No cleaved caspase-3 was detected in mt-p53 J82 and TCCSUP cells.

To determine the effects and mechanisms of anthracyclines-induced apoptosis in human TCC cells, we measured the activation of caspase-3/7 and cleavage of PARP in RT4, SW780, 5637, UM-UC-3, T-24, J82 and TCCSUP cells 24 hours after treatment with 1 µM Dox, 10 µM AD 312, and 1 µM AD 198. Caspase-3/7 activities were significantly increased in both tested wt-p53 cells by 6.7-, 4.2-, and 7.8-fold in RT4 cells and 3.6-, 1.6-, and 5.8-fold in SW780 by Dox, AD 312, and AD 198, respectively, as shown in Figure 3A. On the other hand, caspase-3/7 activities were only moderately upregulated in the mt-p53 cells by 1.7-, 1.4-, and 3.4-fold in 5637 cells and 1.2-, 1.0-, and 1.7-fold in J82 cells by Dox, AD 312, and AD 198 treatments, respectively as shown in Figure 3A. In UM-UC-3 and T-24 cells, caspase-3/7 activity was highly upregulated by 7.3-, and 5.0-fold, respectively by Dox treatment, and only 1.6- and 1.7- fold, respectively in UM-UC-3 cells and by 1.8-, and 2.0-fold, respectively in T-24 cells by AD 312 and AD 198, respectively, as shown in Figure 3A. Caspase 3/7 activity in TCCSUP cells were unchanged by any drug treatments as shown in Figure 3A. The cleavage of the caspase-3 protein by Dox, AD 312, and AD 198 in RT4, SW780, 5637, UM-UC-3, T-24, J82, and TCCSUP cells was confirmed by WB analysis as shown in Figure 3B. Dox, AD 312, and AD 198 treatments increased cleaved caspase-3 in RT4, SW780, and 5637 cells (Figure 3B). Dox, but not AD 312 or AD 198 treatment increased cleavage of caspase-3 in UM-UC-3 and T-24 cells. No cleavage of caspase-3 was detected by any anthracycline treatments in mt-p53 J82 and TCCSUP cells as shown in Figure 3B.

**Figure 4:**
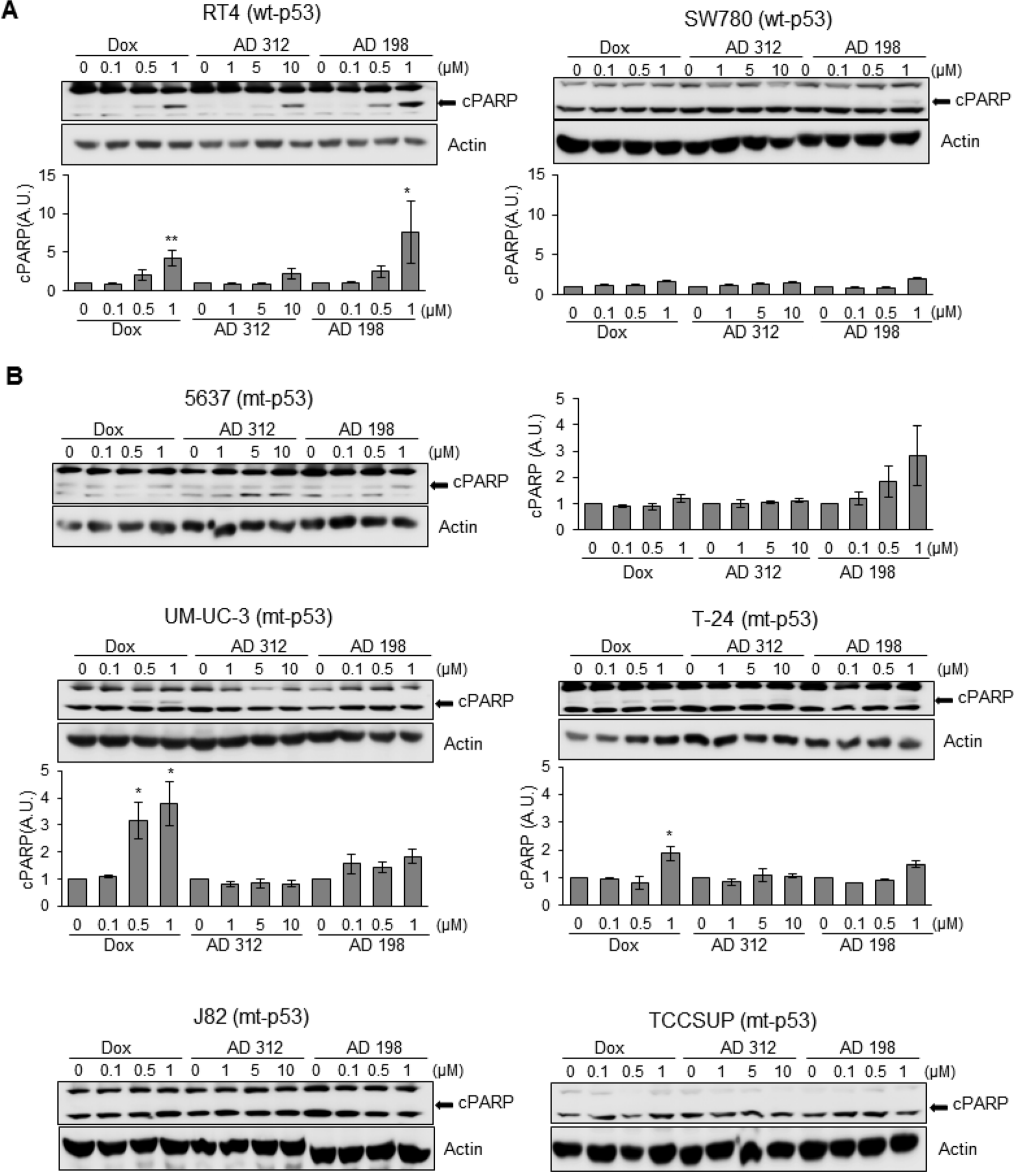
Dox, AD 312, and AD 198 treatments increased cleaved PARP (cPARP) in wt-p53 bladder TCC cells (**A**) The wt-p53 and (**B**) mt-p53 bladder TCC cells were treated with 0.1 µM, 0.5 µM, and 1 µM of Dox or AD 198 or 1 µM, 5 µM, and 10 µM of AD 312 in serum free media for 24 hours and cPARP expression was detected by WB analysis. Actin was used as a loading control. The increased cPARP expression was detected after high doses of all drug treatments in RT4 cells and after high dose of AD 198 in SW780 cells. Antracycline treatments did not change the levels of cPARP protein in 5637 cells. Dox treatment upregulated cPARP in UM-UC-3 and T-24 cells and no cPARP was detected in J82 and TCCSUP cells. Densitometry analysis of normalized cPARP protein levels to actin and relative to the DMSO-treated (control) groups are shown as means ± S.E. of three readings of two independent experiments. Statistical analyses were performed using the Student’s two tailed paired *t*-test and significance was determined at ^*^p ≤ 0.05 and ^**^p ≤ 0.01.

The poly (ADP-ribose) polymerase (PARP) family of proteins, is cleaved by caspases at the initiation of apoptosis. Dox (1 µM), AD 312 (10 µM), and AD 198 (1 µM) increased PARP cleavage in wt-p53 RT4 cells as shown in Figure 4A that was consistent with the upregulated caspase 3/7 activity and cleaved caspase-3 protein expression (Figure 3). Likewise, AD 198 (1 µM) increased PARP cleavage in SW780 (Figure 4A) and 5637 cells (Figure 4B). Dox treatments (0.5 µM and 1 µM) significantly (^*^p ≤ 0.05) increased cleavage of PARP in mt-p53 UM-UC-3 and T-24 cells (Figure 4B) that was consistent with the increased caspase 3/7 activities and cleaved caspase-3 expression. The expression of cleaved PARP was not detected in mt-p53 J82 or TCCSUP cells (Figure 4B).

### siRNA p53 blocks Dox-, AD 312-, and AD 198-induced apoptosis in wt-p53 RT4 cells

**Figure 5:**
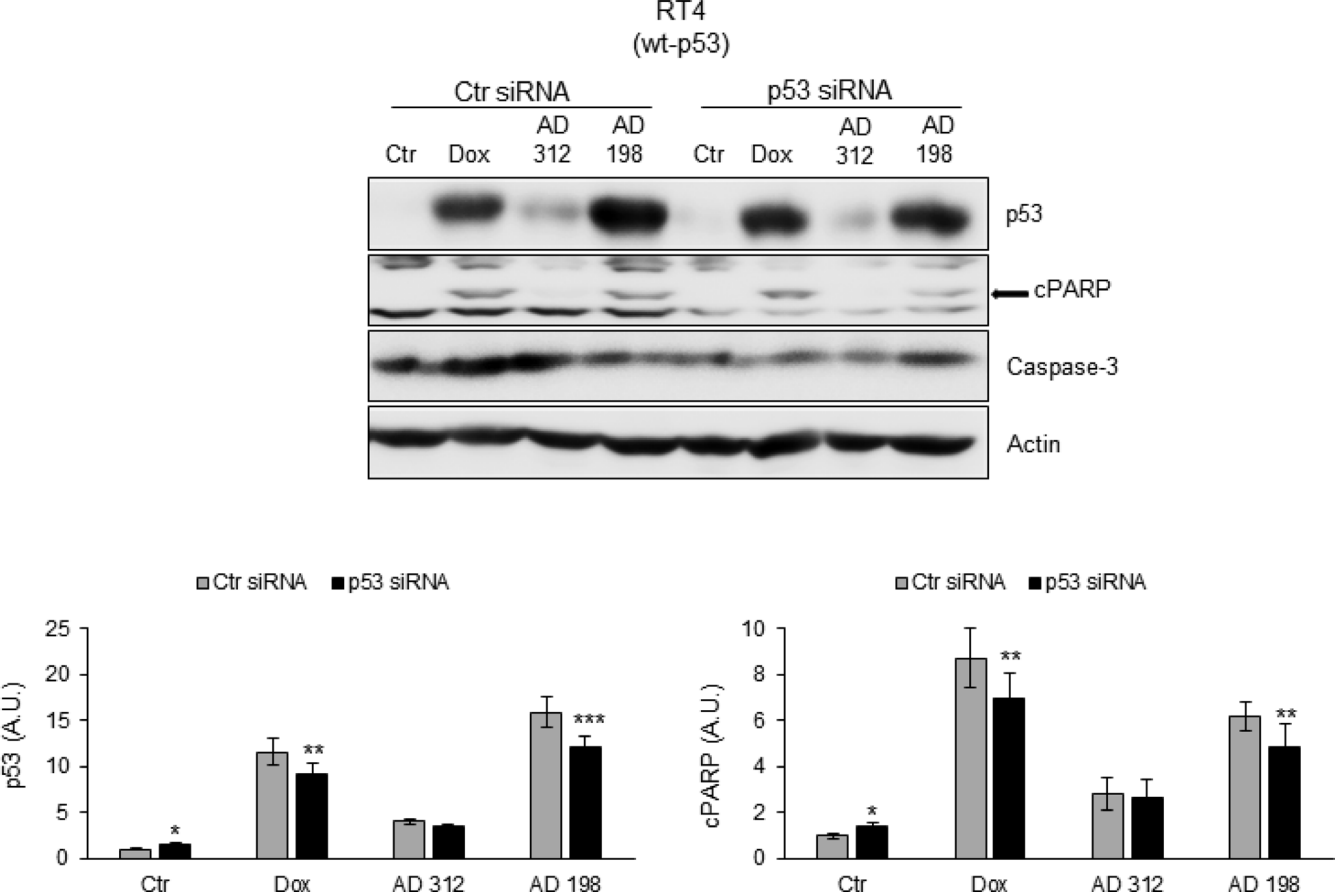
p53 siRNA blocked Dox-, AD 312-, and AD 198-induced cPARP in wt-p53 RT4 cells The wt-p53 bladder TCC RT4 cells were transfected with 75 nM p53 siRNA and control siRNA. Twenty-four hours after transfection, the cells were treated with DMSO (control), Dox (1 µM), AD 312 (10 µM), or AD 198 (1 µM) in serum free media for an additional 24 hours. The expression levels of p53 and cPARP were detected by WB analysis. Actin was used as a loading control. p53 siRNA transfection blocked Dox-, AD 312-, and AD 198-induced cPARP in wt-p53 bladder TCC RT4 cells. Densitometry analysis of normalized p53 or cPARP protein levels to actin and relative to the control-treated (DMSO) groups are shown as means ± S.E. of three readings of three independent experiments. Statistical analyses were performed using the Student’s two tailed paired *t*-test and significance was determined at ^*^p ≤ 0.05, ^**^p ≤ 0.01 and ^***^p ≤ 0.001.

To confirm that Dox-, AD 312-, and AD 198-induced apoptosis through PARP cleavage is p53-dependent, RT4 cells were transfected with p53 siRNA. As shown in Figure 5, p53 siRNA transfection significantly inhibited Dox-, AD 312-, and AD 198-induced cleavage of PARP in wt-p53 RT4 cells.

### PRIMA-1 inhibited cell viability in the anthracycline-resistant mt-p53 J82 cells, but not in wt-p53 RT4 cells

**Figure 6:**
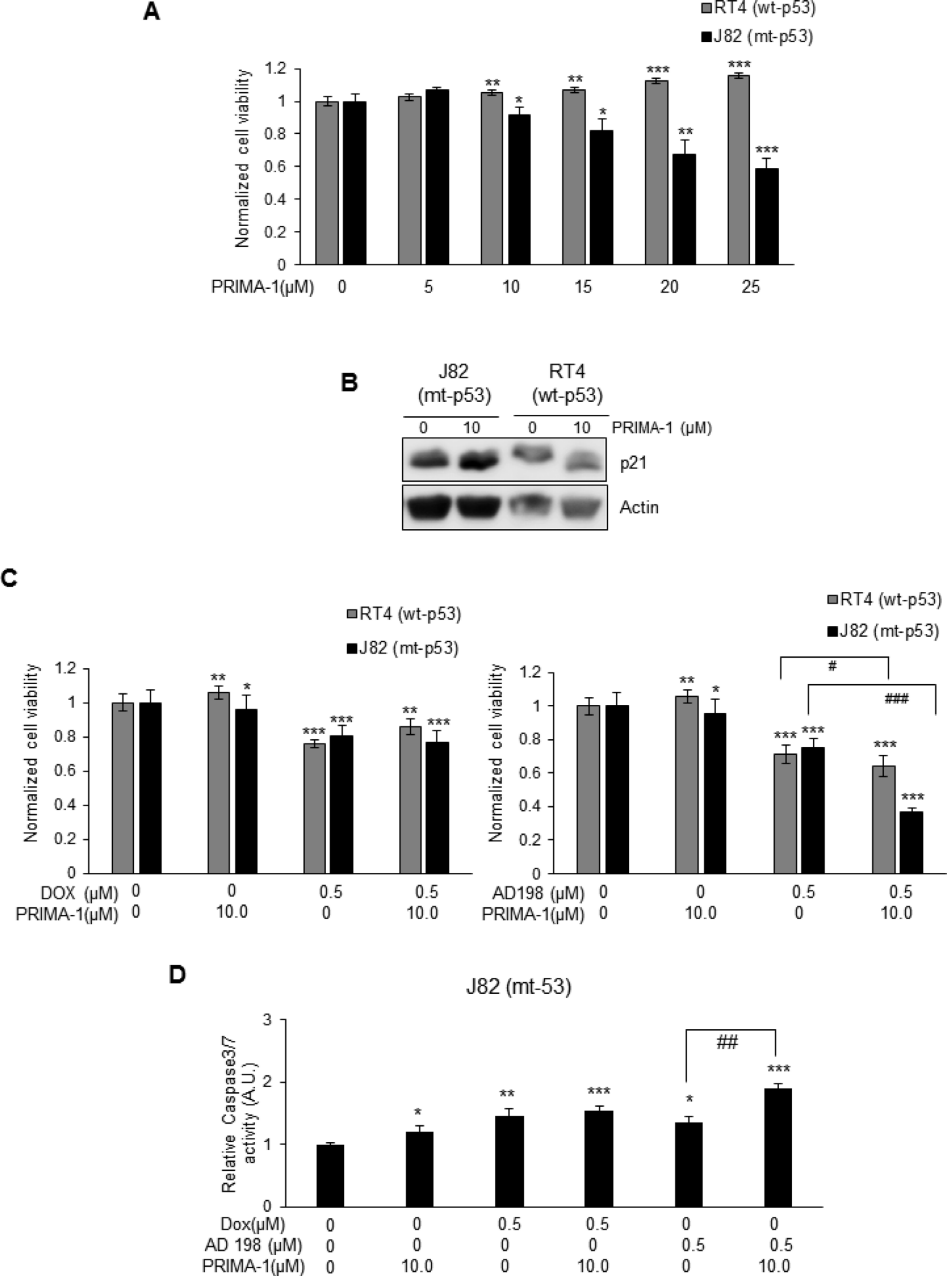
PRIMA-1 sensitized mt-p53 J82, but not wt-p53 RT4 cells to AD 198 treatment (**A**) wt-p53 RT4 and mt-p53 J82 cells were treated with increasing doses of PRIMA-1 (5, 10, 15, 20, and 25 µM) for 48 hours and cell viability was assessed by MTS assay. PRIMA-1 treatments did not affect the cell viability of wt-p53 RT4 cells. On the other hand, PRIMA-1 treatment decreased cell viability in a dose-dependent manner in mt-p53 J82 cells. (**B**) PRIMA-1 treatment (10 µM) upregulated p21 protein levels in mt-p53 J82 cells, but not in wt-p53 RT4 cells. (**C**) RT4 and J82 cells were treated with 10 µM PRIMA-1 alone or in combination with 0.5 µM of Dox (left panel) or AD 198 (right panel) for 48 hours and cell viability was assessed by MTS assay. A co-treatment of AD 198 with PRIMA-1 highly significantly decreased cell viability as compared to AD 198 treatment alone in mt-p53 J82 cells (^###^*p* ≤ 0.001). Values shown as means **±** S.E. of four replicates of two independent experiments of normalized cell viability of treated groups to control (DMSO) group. (**D**) The activity of caspase 3/7 in mt-p53 J82 cells were measured by Caspase-Glo3/7 luminescence assay after treatments. Co-treatment of AD 198 with PRIMA-1 increased activities of caspase-3/7. Data shown here are normalized caspase activities of drug-treated groups to control groups as means ± S.E. of three replicates of two independent experiments. Statistical analyses were performed using the Student’s two tailed paired *t*-test and significance was determined comparing treatment to control groups (^*^p < 0.05, ^**^p ≤ 0.01, and ^***^p ≤ 0.001) and comparing anthracycline treatments alone to their combination with PRIMA-1 treatment (^#^*p* ≤ 0.05, ^##^*p* ≤ 0.01, and ^###^*p* ≤ 0.001).

Two human TCC cell lines, RT4 (wt-p53 anthracycline-sensitive cell line) and J82 (mt-p53 anthracycline-resistant cell line), were treated with 5, 10, 15, 20, and 25 µM of PRIMA-1 for 48 hours to restore p53 protein function. PRIMA-1 did not have any effect on cell viability in wt-p53 RT4 cells as shown in Figure 6A. On the other hand, PRIMA-1 decreased cell viability in a dose-dependent manner in mt-p53 J82 cells (Figure 6A) by restoring function of p53 through increased p21 expression that is a downstream target of p53 (Figure 6B). PRIMA-1 did not affect levels of p21 in wt-p53 RT4 cells. Calculated IC_50_ values of PRIMA-1 was 31.6 µM in J82 cells.

Co-treatment of 10 µM PRIMA-1 with 0.5 µM Dox decreased cell viability of wt-p53 RT4 cells by 14% and co-treatment of PRIMA-1 with 0.5 µM AD 198 decreased cell viability by 36% (^#^*p* ≤ 0.05) as shown in Figure 6C. In contrast, a strong synergistic effect by co-treatment of 10 µM PRIMA-1 with 0.5 µM AD 198 was detected in mt-p53 J82 cells. Treatments by 10 µM PRIMA-1, 0.5 µM Dox, and 0.5 µM AD 198 alone inhibited cell viability of J82 cells by 4%, 20%, and 25%, respectively, however, co-treatment of PRIMA-1 with 0.5 µM AD 198 significantly (^***^p ≤ 0.001) inhibited cell viability by 63% in mt-p53 J82 cells as shown in Figure 6C. This synergistic effect was not detected by co-treatment of Dox with PRIMA-1 in J82 cells (20% by 0.5 µM Dox alone vs 23% by co-treatment) (Figure 6C). Similar results were also obtained by co-treatment of PRIMA-1 with AD 198 in another mt-p53 bladder TCC 5637 cells as shown in Supplementary Figure 2. A high dose of PRIMA-1 (50 µM) was toxic to both mt-p53 J82 and 5637 cells as shown in Supplementary Figure 2A and 2B.

To determine the mechanisms of PRIMA-1 and Dox or AD 198 co-treatment-induced inhibition of cell viability in mt-p53 J82 cells, levels of cPARP were evaluated by WB analysis as shown in Supplementary Figure 3. Co-treatment of PRIMA-1 with Dox or AD 198 did not affect the expression of cPARP in RT4 cells. No levels of cPARP were detected in J82 cells after PRIMA-1 treatment or in combination with anthracyclines due to low activity of caspase3/7 as shown in Supplementary Figure 3. Co-treatment of PRIMA-1 (10 µM) with AD 198 (0.5 µM) significantly (^##^*p* ≤ 0.01) increased caspase 3/7 activities by 1.4-fold as compared to AD 198 treatment alone in mt-p53 J82 cells (Figure 6D).

### AD 198 treatment decreased levels of oncoprotein c-myc in mt-p53 bladder cancer cells

**Figure 7:**
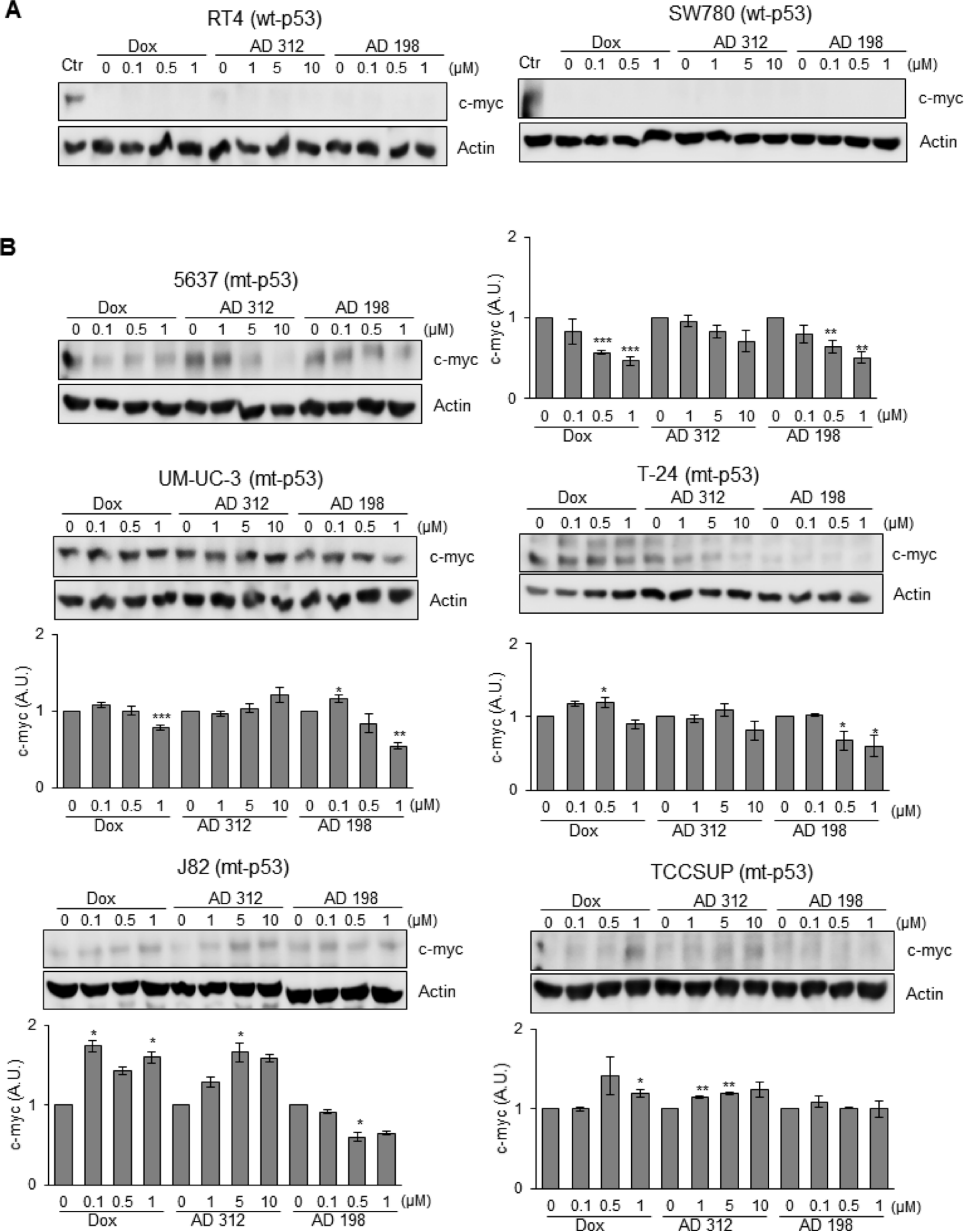
AD 198 decreased c-myc protein levels in mt-p53 bladder TCC cells Human bladder TCC cells were treated with 0.1 µM, 0.5 µM, and 1 µM of Dox or AD 198 or 1 µM, 5 µM, and 10 µM of AD 312 in serum free media for 24 hours and levels of c-myc protein expression were detected by WB analysis. Actin was used as a loading control. (**A**) No levels of c-myc protein expression were detected in the wt-p53 RT4 and SW780 cells after anthracycline treatments. UM-UC-3 cells (lane 1) expressing c-myc protein were used as a positive control. (**B**) Significant decrease of c-myc protein expression was detected in the mt-p53 5637, UM-UC-3, T-24, and J82 cells after AD 198 treatment in a dose-dependent manner. Densitometry analysis of normalized c-myc protein levels to actin followed by normalization of anthracyclines treated to control groups are shown in chart as means ± S.E. of three readings of two independent experiments. Statistical analyses were performed using the Student’s two tailed paired *t*-test and significance was determined at ^*^p ≤ 0.05, ^**^p ≤ 0.01, and ^***^p ≤ 0.001.

The basal levels of c-myc expression were not detected in wt-p53 RT4 and SW780 cells as shown in Figure 7A. On the other hand, endogenous levels of c-myc were detected in all five tested mt-p53 cell lines; 5637, UM-UC-3, T-24, J82, and TCCSUP as shown in Figure 7B. AD 198 treatment suppressed c-myc expression in all tested mt-p53 cells, except TCCSUP cells. Dox treatment suppressed c-myc expression only in mt-p53 5637 and UM-UC-3 cells as shown in Figure 7B.

## DISCUSSION

AD 312 and AD 198 are novel bifunctional Dox-derived anthracyclines [11]. Bifunctional compounds offer unique biochemical mechanisms to inhibit growth of tumor cells resulting in treatment that is more efficient. In contrast to Dox, AD 312 and AD 198 are non-cardiotoxic [11]. AD 312, like Dox, acts in the nucleus of cells as a topoisomerase II inhibitor, while AD 198 localizes in the cytoplasm to activate the PKC signaling pathway [8]. Our study focused on validating the efficacy of Dox, AD 312, and AD 198 in human bladder cancer cell lines with respect to p53 mutation status. Our results demonstrate that tested anthracyclines decreased bladder cancer cell viability and induced apoptosis *in vitro*, and *TP53* mutational status played a critical role. Tested anthracyclines significantly activated caspase 3/7 and cleaved PARP in wt-p53 RT4 and SW780 cells. However, anthracyclines did not induce apoptosis in high grade drug-resistant J82, and TCCSUP cells that have mutations of p53 in the TMD. Furthermore, the anthracyclines-induced cleavage of PARP was blocked by p53 siRNA in wt-p53 RT4 cells. Restoration of p53 protein function by PRIMA-1 co-treatment in anthracycline-resistant mt-p53 J82 cells decreased cell viability and further sensitized cells to AD 198 treatment.

The tumor suppressor gene *TP53* mediates cellular responses to different stimuli, including DNA damage, stress, oncogene activation, and hypoxia [38]. Mutations in the *TP53* gene are found in approximately 0–14% of low grade and 24–56% of high grade muscle invasive bladder cancer patients [39]. Overexpression and mutations of *TP53* is associated with increased drug- and radio-resistance and is an adverse prognostic marker for patients diagnosed with bladder cancer [23, 40] and with lymphoma [41]. The *TP53* mutation status is well-characterized in human TCC cell lines as shown in Table 1 [42, 43]. RT4 and SW780 have wild-type *TP53*, while *TP53* mutations are reported in 5637, UM-UC-3, T-24, J82, and TCCSUP cell lines [42, 43]. Treatment by Dox, AD 312 and AD 198 decreased cell viability of tested wt- and mt-p53 bladder TCC cells as shown in Figure 1 and Table 2. Based on calculated IC_50_ values, wt-p53 RT4 and SW780 cells and mt-p53 5637, UM-UC-3, and T-24 cells responded better to the anthracycline treatments than high grade mt-p53 J82 and TCCSUP cells with *TP53* mutations in the TMD. In this diverse range of different mutations present in tested seven bladder cancer cell lines, the observed diversity in responses to anthracycline treatments might be caused by the certain *TP53* mutations. The function of known *TP53* mutations in cancer is still not fully characterized. The *TP53* mutations are located in all coding exons of *TP53* gene, but predominantly in exons 4–9 of chromosome 17, which encode the DNA-binding domain of p53 protein [30, 42–44]. In our study, we have tested the effects of anthracyclines in five mt-p53 bladder cancer cells that had different mutations of p53. 5637 cells have the R280T mutation of p53 [42, 43] that has been shown to promote proliferation of human glioma cells [45] (reviewed in [30]). The G245S mutation of p53 detected in 5637 cells was reported by Bamford *et al.*, [46] which has been shown to promote the epithelial-to-mesenchymal transition in MCF10-A cells [47] (reviewed in [30]). We have no information about the role of mutated p53 described in T-24 (Y126) or UM-UC-3 (F113C) cells [42, 43]. J82 cells have the E271K, V274F, and K320N mutations as described previously [42, 43]. As reported by Liu *et al.*, [48] lysine 320 is acetylated in response to DNA damage, which modulates p53 DNA binding. The mutation of V274F of p53, expressed also in J82 cells [42, 43], plays an important role in the development of drug-resistance and avoidance of cell death as shown in prostate PC-3 and DU145 cells [49, 50] (reviewed in [30]). The nonsense mutation found in the TMD, at codon 349 of exon 10 in p53 results in the truncation of the p53 protein in TCCSUP cells [43]. Truncation of p53 protein at codon 369 of exon 10 has been demonstrated to cause cytoplasmic retention and loss of transactivation function in neuroblastoma SK-N-AS cells [51]. These p53 mutations in J82 and TCCSUP cells are most likely responsible for the anthracyclines-resistance as compared to other tested wt- and mt-p53 bladder TCC cells. Based on our results, we conclude that mutations of the p53 TMD may play a critical role in anthracycline-induced cell apoptosis in drug-resistant mt-p53 J82 and TCCSUP cells.

AD 198 was the most effective from tested anthracyclines in inhibiting cell viability of bladder cancer cells as shown in Figure 1 and Table 2. Previously published studies also demonstrate that AD 198 is superior to Dox treatment in inhibiting cell viability of human bladder cancer cells T-24 and UM-UC-3 [52], human oral squamous cell carcinoma cells SCC-25 and 1483 [53], canine bladder TCC cells K9TCC#1-Lillie, K9TCC#2-Dakota, and K9TCC#4-Molly, and canine osteosarcoma cells K9OSA#1-Zoe, K9OSA#2-Nashville, and K9OSA#3-JJ [18]. Based on IC_50_ values, AD 312 was the least effective in inhibiting viability of bladder TCC cells as shown in Figure 1 and Table 2. A study by Glaves *et al.*, [54] reported that AD 312 is less effective than Dox in inhibiting cell growth of the human bladder TCC BL13 cells *in vitro*. However, *in vivo* results from the same study demonstrated that AD 312 inhibited tumor growth more effectively than Dox in the BL13 xenograft mouse model, suggesting that the bifunctional activity of AD 312 resulted in a better responses *in vivo* [54]. More importantly, AD 312 is less toxic for a long-term usage compared to Dox *in vivo*, suggesting that despite required higher doses of AD 312 for an effective treatment response, AD 312 might be a better option for the patients’ treatment than Dox [54].

P53 plays a major role in cell-cycle regulation and induction of apoptosis and its levels are elevated in response to apoptotic stimuli, such as induced DNA damage by treatments with chemotherapeutic drugs. The anthracycline treatments significantly increased p53 levels in a dose-dependent manner in wt-p53 RT4 and SW 780 cells (Figure 2A). In contrast, neither Dox, AD 312, nor AD 198 treatments significantly altered p53 levels in mt-p53 5637, UM-UC-3, J82, T-24, and TCCSUP cells and (Figure 2B). Our findings are in agreement with a study by Hinata *et al.* [55], where radiation-induced apoptosis was also impacted by the mutation status of *TP53* in bladder TCC cells. Radiation-induced *TP53* mRNA expression in wt-p53 KK47 and RT4 cells, while mRNA levels of *TP53* were not elevated in mt-p53 T-24, 5637, and UM-UC-3 cells [55]. A treatment with flavokawain A, a phytochemical with potential anti-tumor activity, induced a G_1_ phase cell-cycle arrest in wt-p53 bladder cancer cells in contrast to induced G_2_ phase cell-cycle arrest in mt-p53 bladder cancer cells [56].

Apoptosis associated with the upregulation of p53 causes rapid activation of caspase-3 protease leading to proteolytic cleavage of PARP. This pathway has been previously demonstrated in C6 glioma cells by cordycepin [57] and andrographolide [58] treatments. Our results are in an agreement with these studies, where a strong correlation between p53 upregulation, caspase 3/7 activation, and PARP cleavage was detected by anthracycline treatments in tested bladder TCC cells as shown in Figures 2–5. Dox and AD 198 treatments significantly increased the expression of p53 protein levels and activated caspase-3/7 in wt-p53 RT4 and SW780 cells (Figures 2–3). In mt-p53 J82 and TCCSUP cells no increase in p53 protein levels, caspase 3/7 activity, and cleaved caspase-3 were detected after anthracycline treatments (Figures 2–3).

To confirm that Dox-, AD 312-, and AD 198-induced cleavage of PARP and caspase 3/7 activity is p53-dependent, wt-p53 RT4 cells were transfected with p53 siRNA (Figure 5). The p53 siRNA transfection blocked anthracyclines-induced cleavage of PARP in treated wt-p53 RT4 cells. To restore p53 function in mt-p53 TCC cells, we tested ability of PRIMA-1 co-treatment [35, 36] to improve the efficacy to anthracycline treatments by restoring p53 function in a drug-resistant high grade J82 cells. Similar to the observations by Bykov *et al.* [35], PRIMA-1-induced cell death in tested bladder TCC cells was dependent on *TP53* mutation status. While PRIMA-1 treatments inhibited cell viability in a dose-dependent manner in mt-p53 J82 cells, PRIMA-1 treatment had no effect on cell viability of wt-p53 RT4 cells (Figure 6). Importantly, sensitivity of J82 cells to AD 198 was significantly increased by a co-treatment of AD 198 (0.5 µM) and PRIMA-1 (10 µM) at lower doses. This suggest that PRIMA-1 sensitizes anthracycline-resistant mt-p53 bladder cancer cells that might be a more effective treatment option for bladder cancer. Treatment of PRIMA-1 restores sensitivity to VMY-1-103 (a CDK inhibitor) through restoration of p53 activity in mt-p53 prostate cancer cells [59]. Additionally, a co-treatment of PRIMA-1 with 3-BrPA (an inhibitor of glycolysis) induces cell death in mt-p53 bladder T-24 cells, but not in wt-p53 RT4 cells [60]. Overall, our data suggest that Dox- and AD 198-induced cell death through caspase-3/7 and PARP cleavage is dependent on mutation of p53.

In order to better understand the response to AD 198 treatment in bladder cancer cells, we studied the expression of oncoprotein c-myc. It has been previously demonstrated that Dox treatment suppresses c-myc expression in MCF-7 breast tumor cells [61]. In our study, we detected that AD 198 suppressed c-myc expression in mt-p53 bladder TCC cells 5637, UM-UC-3, T-24, and J82, while no endogenous c-myc expression was observed in the wt-p53 TCC cells RT4 and SW780. Consistent with our findings, AD 198 treatment also suppressed c-myc expression in multiple myeloma and lymphoma cells [19]. AD 198 has better efficacy in inhibiting cell viability compared to Dox and AD 312 in mt-p53 bladder TCC cells, which could be attributed through suppression of c-myc protein expression. While a previous study reported that c-myc is required for the induction of apoptosis by etoposide and Dox treatments in HO15.19 cells, a c-myc negative rat fibroblasts [62], our results contradict those findings. Dox, AD 312, and AD 198 induced apoptosis in both c-myc positive and negative bladder TCC cells.

In conclusion, tested anthracyclines Dox, AD 312, and AD 198 suppressed cell viability in human bladder TCC cells (RT4, SW780, 5637, UM-UC-3, T-24, J82, and TCCSUP). In this study, AD 198 was more potent than Dox and AD 312 in suppressing cell viability in tested TCC cells, except UM-UC-3. Dox, AD 312, and AD 198 treatments significantly increased levels of the tumor suppressor protein p53 in wt-p53 RT4 and SW780 cells, while no considerable changes in p53 levels were observed in mt-p53 5637, UM-UC-3, T-24, J82, and TCCSUP cells. Dox, AD 312, and AD 198 treatments increased caspase-3/7 activities and PARP cleavage in wt-p53 cells, but not in mt-p53 TCC cells. Additionally, AD 198 decreased the levels of the oncoprotein c-myc, suggesting its novel mechanism of actions in suppressing bladder TCC cell growth. Co-treatment of PRIMA-1 improved the potency of AD 198 in inhibiting the cell viability by restoring p53 function in high grade mt-p53 bladder TCC cells. Restoration of p53 function was demonstrated by increased expression of p21 protein in J82 cells. The p53 siRNA transfection blocked anthracyclines-induced cleavage of PARP in treated wt-p53 bladder TCC cells. In conclusion, our results demonstrated that the anthracycline-induced resistance in tested bladder cancer cells detected by MTS assay and caspase 3/7 activities positively correlated with *TP53* mutations in the TMD occurring in mt-p53 J82 and TCCSUP cells. Further, AD 312 and AD 198 are promising new chemotherapies for bladder cancer, especially in combination with PRIMA-1 that sensitized mt-p53 cells to AD 198 treatment.

## MATERIALS AND METHODS

### Reagents and antibodies

Unless otherwise stated, all reagents and media were purchased from Fisher Scientific (Pittsburgh, PA, USA). Doxorubicin (Dox) and PRIMA-1 were purchased from Sigma-Aldrich (St. Louis, MO, USA). The control and p53 siRNA were purchased from Cell Signaling Technology (Boston, MA, USA). *Trans*IT-TKO^®^ transfection reagent was purchased from Mirus Bio LLC (Madison, WI, USA). AD 312 and AD 198 were kindly provided by Dr. Leonard Lothstein, The University of Tennessee, Health Science Center in Memphis, TN, USA. Antibodies for PARP (Catalog #9542), c-myc (D84C12, Catalog #5605), and cleaved caspase-3 (Asp175, Catalog #9661) were purchased from Cell Signaling Technology (Boston, MA, USA). Antibodies for p53 (Bp53-12, sc-263), total caspase-3 (H-277, sc-7148), p21 (C-19, sc-397), and actin (C-11, sc-1615) were purchased from Santa Cruz Biotechnology (Santa Cruz, CA, USA). Secondary anti-rabbit (Catalog #7074) and anti-mouse (Catalog #7076) antibodies were purchased from Cell Signaling Technology (Boston, MA, USA).

### Human cell lines

Wild-type p53 (wt-p53) human bladder TCC cell lines RT4 (Grade I) and SW780 (Grade I), and mutated p53 (mt-p53) human bladder 5637 (Grade I), UM-UC-3 (Grade III), J82 (Grade III), T-24 (Grade III), and TCCSUP (Grade IV) TCC cell lines were purchased from ATCC (Manassas, VA) [63]. Human RT4 and T-24 cells were maintained in McCoy’s media, SW780, and 5637 cells were maintained in RPMI-1640 media, UM-UC-3 cells were maintained in MEM media, and J82 and TCCSUP cells were maintained in MEM media supplemented with non-essential amino acids and sodium pyruvate. All media were supplemented with 10% FBS (Atlanta Biologicals Flowery Branch, GA, USA), 100 I.U. penicillin, and 100 µg/ml streptomycin and grown in an atmosphere of 5% CO_2_ at 37° C. The UM-UC-3 and T-24 cell lines were authenticated via short-tandem repeat (STR) DNA profiling by Genetica DNA laboratories (Burlington, NC, USA). Other cell lines obtained from ATCC were used in this study for fewer than 6 months after resuscitation. A detailed description of *TP53* mutation status in each cell line is described in Table 1.

### MTS assay

Cells were seeded at a density of 5,000 cells/well in complete media in 96-well tissue culture plates in four replicates and allowed to attach for 24 hours. Cells were then treated with Dox, AD 312, AD 198, and PRIMA-1 in a dose-dependent manner in complete media for an additional 48 hours. For co-treatment of anthracyclines with PRIMA-1, cells were treated with both drugs simultaneously in complete media for 48 hours. MTS reagent (Promega Corporation, Fitchburg, WI) was added to each well and allowed to incubate for an hour at 37° C. The absorbance was measured at 490 nm using a multi-mode microplate reader (Bio-Tek Instruments, Inc., Winooski, VT). Values presented in Figures are a mean ± S.E. of normalized cell viability of treated to the control (DMSO) groups from four replicates of at least two independent experiments. IC_50_ values were calculated by plotting a fitted dose response curve and using the equation y = mx + c; where y = 0.5, m is slope, and c is the y-intercept. IC_50_ values for each drug and for each cell line is presented in Table 2.

### Western blot

Cells were seeded at a density of 2 × 10^6^ cells per 10 cm petri dish in complete media for 24 hours to allow them to attach and followed by the drug treatments at the respective concentrations in serum free media for an additional 24 hours. After treatment, protein lysates were harvested, and protein concentrations were quantified using the Pierce^®^ BCA protein assay. An equal amount of proteins (40–60 µg) were loaded on to SDS-PAGE gels and transferred to a nitrocellulose membrane and probed with primary and secondary antibodies. The immunoreactive bands were visualized using the enhanced chemiluminescence system (GE Healthcare Bio-Sciences, Pittsburgh, PA, USA) and images were acquired using the UVP Biospectrum815 imaging system (UVP, Upland, CA). Densitometry analyses were performed using the VisionWorks^©^ software (UVP) and values of treatments were normalized to controls (DMSO) and plotted.

### Caspase-3/7 assay

Twenty-five micrograms of protein lysates harvested from Dox (1µM), AD 312 (10 µM), AD 198 (1 µM), and DMSO (control) treated cells were mixed with Caspase-Glo 3/7 substrate (Promega Corporation) according to the manufacturer’s instructions and incubated at room temperature for an hour. For PRIMA-1 and anthracyclines co-treatment experiments in J82 cells, 60 µg of protein lysates were used for this assay. The luminescence was measured using a Bio-Tek microplate reader (Bio-Tek Instruments, Inc.). Data shown here represent values as a mean ± S.E. of three replicates from two independent experiments obtained from drug-treated groups normalized to the DMSO-treated control group.

### siRNA transfection

For RNA interference of p53, RT4 cells were transfected with 75 nM p53 siRNA or control siRNA using *Trans*IT-TKO transfection reagent (Mirus Bio LLC, Madison, WI, USA) according to the manufacturer’s instructions as published previously [64]. Twenty-four hours after transfection, the cells were treated with vehicle (DMSO, control), Dox (1 µM), AD 312 (1 µM), or AD 198 (1 µM) in serum free media for an additional 24 hours.

### Statistical analysis

Statistical analyses were performed using the Student’s two tailed paired *t*-test and significance was determined at ^*,#^*p* ≤ 0.05, ^**,##^*p* ≤ 0.01, and ^***,###^*p* ≤ 0.001.

## SUPPLEMENTARY MATERIALS FIGURES



## Abbreviations

AD 198: N- benzyladriamycin-14-valerate; Benzarubicin^®^
AD 312: *N*-Nitrosureidodaunorubicin; Daunomustine^®^
ATF2: activating transcription factor 2
CREB: cAMP response element binding protein
Dox: Doxorubicin
mt-p53: mutant p53
p38 MAPK: p38 mitogen-activated protein kinase
cPARP: cleaved poly (ADP-ribose) polymerase
PKC: protein kinase C
PRIMA-1: P53 Reactivation and Induction of Massive Apoptosis-1
TCC: Transitional cell carcinoma
wt-p53: wild type p53
