# Supplementary material for: Mutations of p53 decrease sensitivity to the anthracycline treatments in bladder cancer cells
Source: Oncotarget. 2018 Jun 19;9(47):28514–31. doi: 10.18632/oncotarget.25530 (PMC6033348; doi:10.18632/oncotarget.25530)
Supplement: Supplementary file 1 [file oncotarget-09-28514-s001.pdf]

Mutations of p53 decrease sensitivity to the anthracycline treatments in bladder cancer cells

SUPPLEMENTARY MATERIALS

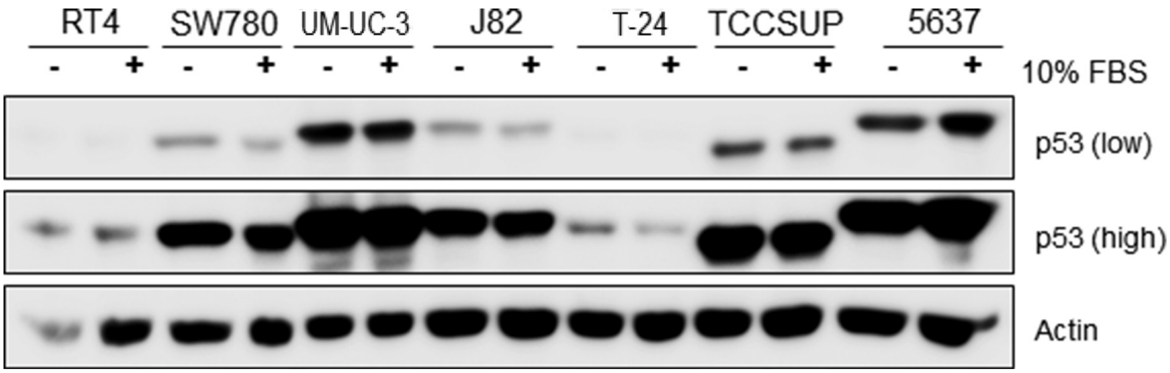

Supplementary Figure 1: P53 expression levels in tested human bladder TCC cells. The bladder TCC cells were treated with and without serum (10% FBS) for 24 hours and expression of p53 was detected by WB analysis. TCCSUP cells have a truncated p53 protein, which was detected at a lower molecular weight. Actin was used as a loading control.

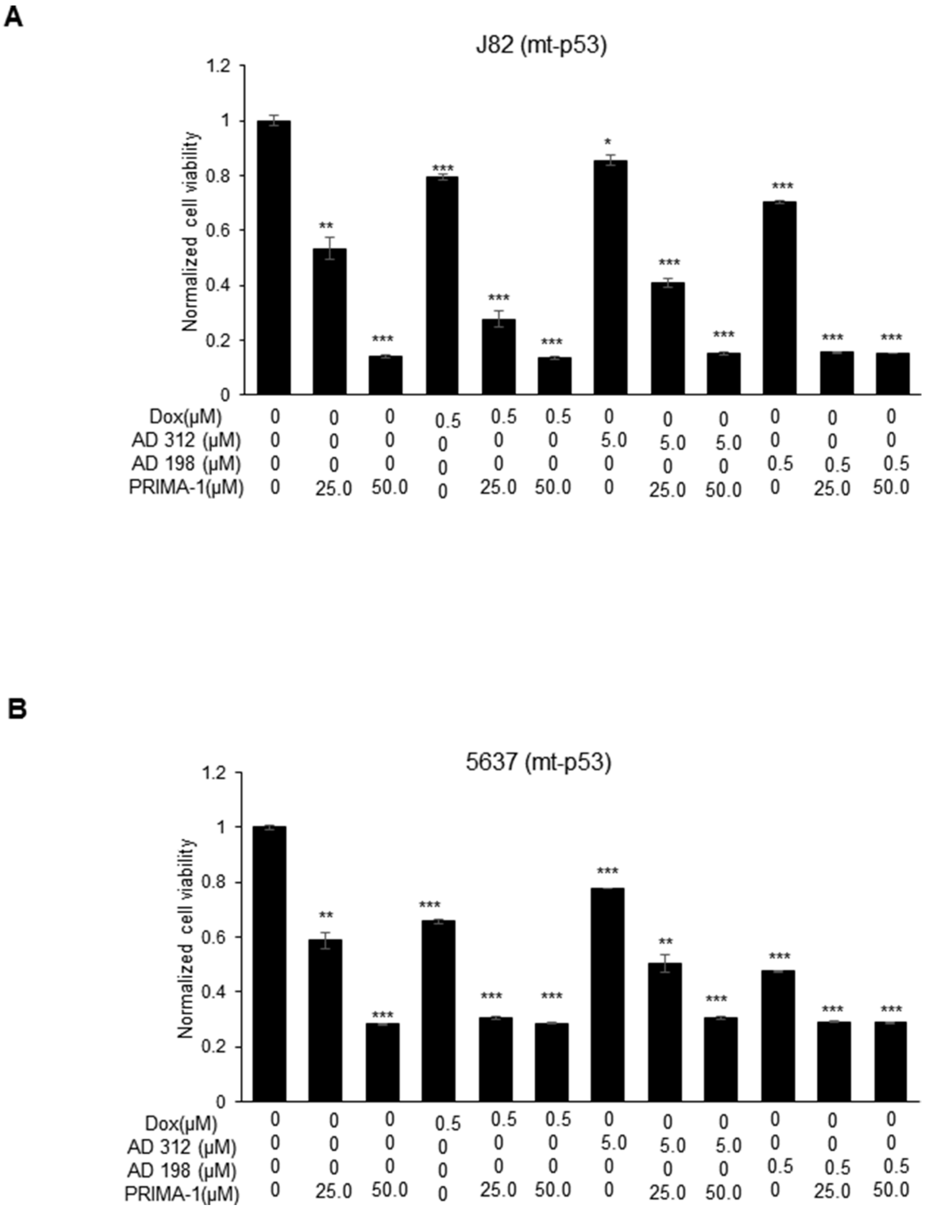

Supplementary Figure 2: PRIMA-1 sensitized mt-p53 J82 and 5637 TCC cells. The mt-p53 bladder TCC cells (**A**) J82 and (**B**) 5637 were treated with 25 μM and 50 μM of PRIMA-1, 0.5 μM Dox, 5 μM AD 312, 0.5 μM AD 198 alone or in combination for 48 hours and cell viability was assessed by MTS assay. PRIMA-1 treatment decreased cell viability in a dose-dependent manner in both tested cells. A co-treatment of 25 μM PRIMA-1 with 0.5 μM Dox, 5 μM AD 312, and 0.5 μM AD 198 significantly ($^{***}p \leq 0.001$) decreased cell viability in both tested cells. Values shown as means ± S.E. of four replicates of normalized cell viability of treated to control groups. Statistical analyses were performed using the Student's two tailed paired *t*-test and significance was determined at $^{*}p \leq 0.05$, $^{**}p \leq 0.01$, and $^{***}p \leq 0.001$.

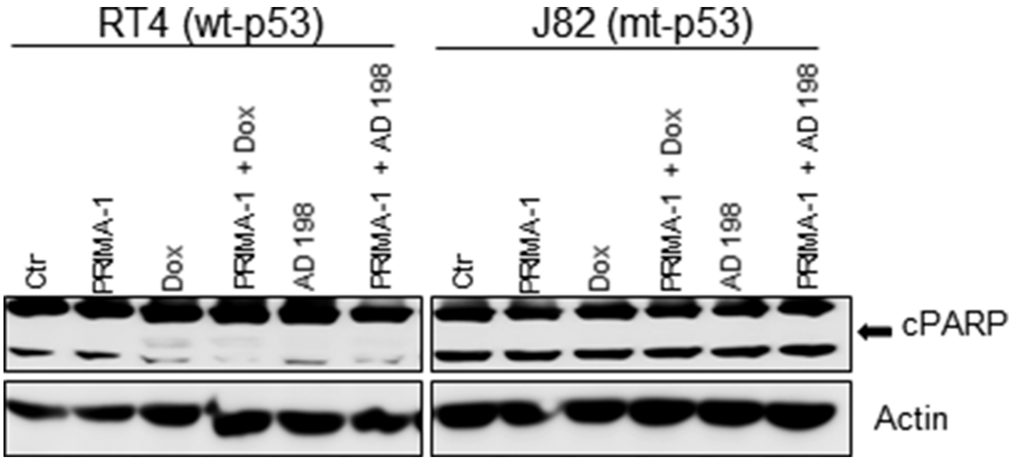

Supplementary Figure 3: Dox-, AD 312-, and AD 198 induced cPARP in wt-p53 bladder TCC RT4 cells. The wt-p53 RT4 and mt-p53 J82 bladder TCC cells were treated with either 10 µM PRIMA-1 or 0.5 µM Dox or 0.5 µM AD 198 alone or in combinations for 24 hours. The expression of the cPARP was detected by WB analysis. The expression of cPARP was detected in wt-p53 RT4 cells, but not in mt-p53 J82 cells. Actin was used as a loading control.
